# Frequency of GJB2 mutations in patients with nonsyndromic hearing loss from an ethnically characterized Brazilian population^☆^

**DOI:** 10.1016/j.bjorl.2017.10.013

**Published:** 2017-11-21

**Authors:** Felippe Felix, Marcia Gonçalves Ribeiro, Shiro Tomita, Mariano Gustavo Zalis

**Affiliations:** Universidade Federal do Rio de Janeiro (UFRJ), Hospital Universitário Clementino Fraga Filho (HUCFF), Rio de Janeiro, RJ, Brazil

**Keywords:** Hearing loss, Deafness, Genetics, Perda de audição, Surdez, Genética

## Abstract

**Introduction:**

In different parts of the world, mutations in the GJB2 gene are associated with nonsyndromic hearing loss, and the homozygous 35delG mutation (p.Gly12Valfs*2) is a major cause of hereditary hearing loss. However, the 35delG mutation is not equally prevalent across ethnicities, making it important to study other mutations, especially in multiethnic countries such as Brazil.

**Objective:**

This study aimed to identify different mutations in the GJB2 gene in patients with severe to profound nonsyndromic sensorineural hearing loss of putative genetic origin, and who were negative or heterozygote for the 35delG mutation.

**Methods:**

Observational study that analyzed 100 ethnically characterized Brazilian patients with nonsyndromic severe to profound sensorineural hearing loss, who were negative or heterozygote for the 35delG mutation. GJB2 mutations were detected by DNA-based sequencing in this population. Participants’ ethnicities were identified as Latin European, Non-Latin European, Jewish, Native, Turkish, Afro-American, Asian and Others.

**Results:**

Sixteen participants were heterozygote for the 35delG mutation; 14 participants, including three 35delG heterozygote's, had nine different alterations in the GJB2 gene. One variant, p.Ser199Glnfs*9, detected in two participants, was previously unreported. Three variants were pathogenic (p.Trp172*, p.Val167Met, and p.Arg75Trp), two were non-pathogenic (p.Val27Ile and p.Ile196Thr), and three variants were indeterminate (p.Met34Thr, p.Arg127Leu, and p.Lys168Arg). Three cases of compound heterozygosity were detected: p.[(Gly12Valfs*2)];[(Trp172*)], p.[(Gly12Valfs*2)](;)[(Met34Thr)], and p.[(Gly12Valfs*2)(;)[(Ser199Glnfs*9)]).

**Conclusion:**

This study detected previously unclassified variants and one case of previously unreported compound heterozygosity.

## Resumo

**Introdução:**

Em diferentes partes do mundo, mutações do gene GJB2 estão associadas a perda auditiva não sindrômica e a mutação homozigótica 35delG (p.Gly12Valfs*2) é uma das principais causas de perda auditiva hereditária. No entanto, a mutação 35delG não é igualmente prevalente em todas as etnias, faz com que seja importante estudar outras mutações, especialmente em países multiétnicos, como o Brasil.

**Objetivo:**

Identificar diferentes mutações no gene GJB2 em pacientes com perda auditiva neurossensorial grave ou profunda não sindrômica de origem genética putativa e negativos ou heterozigotos para a mutação 35delG.

**Método:**

Estudo observacional que analisou 100 pacientes brasileiros caracterizados etnicamente, com perda auditiva neurossensorial grave ou profunda não sindrômica, negativos ou heterozigotos para a mutação 35delG. As mutações de GJB2 foram detectadas por sequenciamento baseado no DNA nessa população. As etnias dos participantes foram identificadas como latino-europeia, não latino-europeia, judaica, nativa, turca, negra, asiática e outras.

**Resultados:**

Dezesseis participantes eram heterozigotos para a mutação 35delG e 14, incluindo três heterozigotos para 35delG, apresentaram nove alterações no gene GJB2. Uma variante, p.Ser199Glnfs*9, detectada em dois participantes, não havia sido relatada anteriormente. Três variantes eram patogênicas (p.Trp172*, p.Val167Met, e p.Arg75Trp), duas não patogênicas (p.Val27Ile e p.Ile196Thr) e três indeterminadas (p.Met34Thr, p.Arg127Leu, e p.Lys168Arg). Três casos de heterozigosidade composta foram detectados: p.[(Gly12Valfs*2)];[(Trp172*)], p.[(Gly12Valfs*2)](;)[(Met34Thr)], e p.[(Gly12Valfs*2)(;)[(Ser199Glnfs*9)]).

**Conclusão:**

Este estudo detectou variantes não classificadas anteriormente e um caso de heterozigosidade composta ainda não relatada.

## Introduction

Hearing loss has a high socioeconomic impact,^1^, ^2^, ^3^ and congenital hearing loss affects about 1/1000 children born in the United States.^4^ Fifty to sixty percent of these cases have genetic origins^5^, ^6^ and, of those, close to 70% are nonsyndromic hearing loss.^1^, ^7^ Mutations in the GJB2 gene encoding the gap junction β-2 protein, connexin 26, are the most common cause of genetic nonsyndromic hearing loss in different parts of the world.^8^ The most common mutation in the GJB2 gene is 35delG, which, in the homozygous state, usually results in severe to profound hearing loss.^9^

However, the 35delG mutation is not equally prevalent across ethnicities. Moreover, when this deletion occurs in the heterozygous state, with monoallelic expression, the origin of hearing impairment cannot be directly attributed to this mutation. Thus, the possibility of other mutations being directly associated with hearing loss should be investigated, especially in multiethnic countries such as Brazil.^10^ DNA-based sequencing of the GJB2 gene may help identify novel mutations associated with hereditary deafness.

This study aimed to identify different mutations in the GJB2 gene in patients with nonsyndromic severe to profound sensorineural hearing loss of putative genetic origin, who were negative or heterozygote for the 35delG mutation. Participating patients received treatment at the Hearing Health Clinic in Otorhinolaryngology Service in a Tertiary University Hospital in Brazil.

## Methods

This study was conducted at a Tertiary University Hospital between September 2011 and August 2014, and was approved by the hospital research ethics committee (043/11). The target populations were patients at the Cochlear Implant Clinic, Otorhinolaryngology Service, with nonsyndromic severe to profound sensorineural/mixed deafness of putative genetic origin, who were negative or heterozygote for the 35delG mutation of the GJB2 gene. Patients with nonsyndromic deafness of putative genetic were those whose clinical, audiological, and imaging (computed tomography and magnetic resonance imaging) examination showed no evidence of other known causes of deafness. Patients or legal guardians who did not agree to sign the informed consent form and/or answer the study questions were excluded. All patients were enrolled for the study by the main author.

Participants’ ethnicities were identified by themselves or by their legal guardians as Latin European, Non-Latin European, Jewish, Native, Turkish, Afro-American, Asian, and Others, following the classification of ethnicity of the Latin-American Collaborative Study of Congenital Malformations (ECLAMC).^11^ The patient or his/her legal guardian were asked if there were other cases of deafness in the family and for the presence of consanguinity.

Of 530 patients seen at the clinic in this period, 100 fulfilled the criteria for participation in the study. All selected participants, or their legal guardians, freely signed the informed consent forms and underwent blood collection for DNA analysis. DNA was extracted from peripheral blood using PureLink™ Genomic DNA kits (Thermo Fisher Scientific, Waltham, MA, USA). After amplification and purification, the genomes were sequenced using the ABI Prism Big-Dye Terminator Cycle Sequencing Kit™ (Applied Biosystems, Foster City, CA, USA) and fragments were assembled using the Sequencing Analysis software version 3.7 (Applied Biosystems), and aligned using the CLC Sequence Viewer 6 software (CLCBio, Aarhus, Denmark) and Mutation Surveyor^®^ software (Softgenetics LLC., State College, PA, USA).

The nomenclature for the description of sequence variants was based on the three-letter code for amino acid sequences preceded by “p.”, following the recommendations of the Human Genome Variation Society (HGVS). Similarly, compound heterozygous sequence variants were also described according to the HGVS nomenclature recommendations. In the case of the 35delG mutation, we opted to use the nomenclature based on nucleotide sequences because it is widely accepted. However, in the description of compound heterozygous 35delG mutations, we used the nomenclature based on amino acid sequences, i.e., p.(Gly12Valfs*2), to follow the HGVS standard nomenclature.

Pathogenicity was determined using the SIFT (Sorting Intolerant from Tolerant) and Polyphen-2 (Polymorphism Phenotyping v2) analysis tools and available information from the following databases: 1000 genomes Project,^12^ Connexin-Deafness Homepage,^13^ and the Deafness Variation Database of the University of Iowa.^14^

The comparison of the variables between the subgroups with and without GJB2 variants was evaluated by Chi-square test.

## Results

The mean age of the study participants was 14.8 years (range: 1–59 years) and 51% of participants were male. Among the 100 participants, 84 lacked the 35delG mutation, and the remaining were heterozygous for 35delG.

Table 1 shows the ethnical characterization of the participants, 67% of whom reported European ancestry, while 57% indicated African descent. No participants reported Jewish, Turkish or Asian ethnicities. There was no statistically significant relationship (*p* = 0.35) between different ethnicities evaluated and the presence of variants of the GJB2 gene (Table 2).

**Table 1:** Ethnicity of patients.

Ethnicity	*N* (*n* = 100)
Latin European	35
Afro-American/Latin European	29
Afro-American	23
Native/Latin European	5
Native	3
Native/Afro-American	3
Non-Latin European	1
Afro-American/Non-Latin European	1

**Table 2:** Ethnicity and presence of GJB2 variants.

Ethnicity	Total (*n* = 100)		With GJB2 variants (*n* = 14)		Without GJB2 variants (*n* = 86)		*p*
	*n*	%	*n*	%	*n*	%	
Latin European	35	35.0	3	21.4	32	37.2	
Afro-American	23	23.0	3	21.4		23.3	
Afro-American/Latin European	29	29.0	7	50.0	22	25.6	0.35
Others	13	13.0	1	7.1	12	14.0	

Of 37 participants who reported a family history of hearing loss, three had a GJB2 gene variant; five reported consanguinity, of which only one had a GJB2 gene variant. As shown in Table 3, 14 participants had GJB2 gene variants other than 35delG – a total of nine variants – including three patients who were heterozygote for the 35delG mutation. Of these other variants, three are pathogenic, two are non-pathogenic, three are as yet indeterminate, and one is a previously undescribed variant. The characteristics of each variant are described in Table 4.

**Table 3:** Profile of participants with sequencing changes.

Case	Variant found	Heterozygous 35delG mutation	Ethnicity	Age (years)	Type of hearing loss
14	p.(Trp172*)	Yes	Afro-American/Latin European	3	Congenital
16	p.[(Val27Ile)(;) [(Arg127Leu)]	No	Afro-American/Latin European	1	Congenital
30	p.(Lys168Arg)	No	Latin European	7	Congenital
35	p.(Val27Ile)	No	Afro-American	5	Congenital
45	p.(Met34Thr)	No	Afro-American	47	Sudden
48	p.(Ser199Glnfs*9)	no	Afro-American/Latin European	4	Congenital
49	p.[(Ile196Thr)(;) [(Lys168Arg)]	No	Afro-American/Latin European	4	Congenital
53	p.(Ser199Glnfs*9)	Yes	Latin European	5	Congenital
62	p.(Val167Met)	No	Afro-American	4	Congenital
64	p.(Lys168Arg)	No	Native/Afro-American	5	Congenital
65	p.(Met34Thr)	Yes	Afro-American/Latin European	47	Progressive
79	p.(Val27Ile)	Yes	Latin European	16	Progressive
82	p.(Arg75Trp)	No	Afro-American/Latin European	3	Congenital
100	p.(Val27Ile)	No	Afro-American/Latin European	2	Congenital

**Table 4:** Variants found in the sequencing of the GJB2 gene.

Variant found and annotation	Description	Effect	SIFT	Polyphen-2	Source*	Type of variant
*Pathogenic*						
p.(Trp172*)	Substitution of a guanine by an adenosine at position 516	Substitution of tryptophan by a stop codon at position 172	Not evaluable	Not evaluable	Da Vinci	Non-sense
p.(Arg75Trp) rs28931 593	Substitution of a cytosine by a thymine at position 223	Substitution of arginine by tryptophan at position 75	0.0	1.0	Da Vinci 1000 genomes Deafness Variation	Missense
p. (Val167Met) rs11103 3360	Substitution of a guanine by an adenosine at nucleotide position 499	Substitution of valine by methionine at position 167	0.04	0.168	Deafness Variation 1000 genomes (possibly pathogenic)	Missense

*Non-pathogenic*						
p.(Val27Ile) rs22740 84	Substitution of a guanine by an adenosine at position 79	Substitution of valine by isoleucine at position 27	0.21	1.0	Da Vinci 1000 genomes Deafness Variation	Missense
p.(Ile196Thr)	Substitution of a cytosine by a thymine at position 587	Substitution of isoleucine by threonine at position 196	0.01	0.922	Benign by Deafness Variation	Missense

Variant found and annotation	Description	Effect	SIFT	Polyphen-2	Source*	Type of variant
*Indeterminate*						
p.(Met34Thr) rs35887622	Substitution of a thymine by a cytosine at position 101	Substitution of methionine by threonine at position 34	0.01	0.38	DaVinci 1000 genomes Deafness Variation	Missense
p.(Lys168Arg) rs2001043 62	Substitution of an adenosine by a guanine at position 503	Substitution da lysine by arginine at position168	0.29	0.074	Benign by 1000 genomes unknown pathogenicity by Deafness Variation	Missense
p.(Arg127Leu) rs1110331 96	Substitution of a guanine by a thymine at nucleotide position 380	Substitution of arginine by leucine at position 127	0.04	0.277	Pathogenic by Deafness Variation benign by 1000 genomes	Missense

*Undescribed*						
p.(Ser199Glnfs*9)	Duplication of the CAGTG segment at position 596	Substitution of serine by glutamine at position 199 and insertion of a frame shift and stop codon	Not evaluable	Not evaluable	Undescribed	Frame shift

## Discussion

Of 100 patients with severe to profound sensorineural hearing loss, 14 had one or more among nine variants in the GJB2 gene other than 35delG, including three who were heterozygote for 35delG. Three of those mutations are pathogenic, one is a previously unreported variant (p.(Ser199Glnfs*9)), and one is a previously unreported case of compound heterozygosity with 35delG, (p.[(Gly12Valfs*2)(;)[(Ser199Glnfs*9)]).

Several studies in different populations have reported the prevalence of the main GJB2 gene variants in patients with deafness.^7^, ^15^ Our study is the first to identify GJB2 variants in an ethnically characterized, profoundly hearing impaired sample from a multiethnic Brazilian population. In addition, we detected a previously unclassified variant and one previously unreported case of compound heterozygosity.

Despite the well-known relationship between ethnicity and mutations of the GJB2 gene,^8^ we did not find a statistically significant relationship between gene changes and ethnicity in the studied group. Apparently, when we split into ethnic groups, the sample size was insufficient to detect significant results.

After 35delG, the p.(Val27Ile) polymorphism was the most prevalent GJB2 variant in the study population, with a frequency of 4.0%. This variant is considered non-pathogenic and has been reported in several studies, both in normal-hearing and hearing-impaired subjects.^16^

The p.(Met34Thr) variant of connexin 26 was first described as dominant pathogenic by Kelsell et al. in 1997.^17^ Later, those findings were questioned and it was suggested that it might be non-pathogenic.^16^, ^18^, ^19^, ^20^, ^21^ In our study population, we found one case, with profound hearing loss, of p.(Met34Thr) in compound heterozygosity with 35delG, p.[(Gly12Valfs*2)](;)[(Met34Thr)]. This presentation was previously documented: a large multicenter study by Snoeckx et al. (2005)^22^ found 38 individuals with sensorineural hearing loss who were compound heterozygotes for p.[(Gly12Valfs*2)](;) [(Met34Thr)] and 16 homozygotes for p.(Met34Thr) who also had hearing impairment. Conversely, Feldmann et al. (2004)^23^ found four normal hearing individuals who were compound heterozygotes for p.[(Gly12Valfs*2)](;)[(Met34Thr)] and characterized this variant as non-pathogenic. The presence of this type of association in normal hearing individuals suggests that this alteration has no pathogenic potential.

One participant in our study had the p.(Trp172*) variant in compound heterozygosity with 35delG (p.[(Gly12Valfs*2)];[(Trp172*)]). The p.(Trp172*) variant, in homozygosity, was first described in a Brazilian individual with bilateral, severe to profound sensorineural hearing loss.^24^ Later, Christiani et al. (2007)^25^ described a cochlear implant recipient with bilateral severe to profound sensorineural hearing loss who was also a compound heterozygote for p.[(Gly12Valfs*2)];[(Trp172*)], like the participant in our study. We genotyped the parents of the patient in our study, both of whom were normal hearing individuals, and found that one parent was heterozygote for 35delG and the other for p.(Trp172*), indicating that these variants may not be pathogenic in heterozygosity.

To date, the p.(Val167Met) variant was detected only in individuals of African ancestry, and only the homozygous form was described as pathogenic.^26^, ^27^, ^28^ In our study, we detected this variant in an Afro-American male participant with congenital deafness, but in heterozygous state. Thus, either the hearing loss of this patient was caused by (unidentified) mutations in a different gene, or p.(Val167Met) is also an autosomal dominant mutation.

We detected the compound heterozygosity p.[(Val27Ile)(;) [(Arg127Leu)] in one of our subjects (n° 16). The p.(Arg127Leu) variant was first described by Tang et al. (2006)^29^ in a heterozygous state in two normal-hearing subjects, one Asian and one Hispanic. However, the low number of cases of this variant thus far reported prevents a definition regarding its pathogenicity. Thus, the origin of hearing loss in case 16 in our study cannot be attributed to these variants either combined with p.(Val27Ile) or in isolation, and may have originated from alteration in another gene.

The p.(Arg75Trp) variant, found in one of our subjects, was first detected in a case of autosomal dominant hearing loss associated with palmoplantar keratoderma, which was characterized as nonsyndromic hearing loss (DFNA3).^30^ However, p.(Arg75Trp) was later detected as a de novo mutation (no family history of hearing loss) in a child with bilateral profound hearing loss and no skin disorders,^31^ exactly as in the case detected in our study.

In our study, three alleles were detected with the p.(Lys168Arg) variant: in one case, it was associated with the p.(Ile196Thr) variant, whereas in the other two cases the heterozygous variant was not associated with other variants. Putcha et al. (2007) detected the p.(Lys168Arg) variant at a frequency of 0.3% (7/1796) in hearing impaired patients.^6^ However, Samanich et al. (2007) detected a heterozygous p.(Lys168Arg) both in patients with sensorineural hearing impairment and in normal hearing controls, and described it as non-pathogenic.^28^ Variant p.(Ile196Thr), found in one patient in compound heterozygosity with p.(Lys168Arg), is a missense variant that cause changes in the amino acid sequences of the last transmembrane domain of connexin 26, but is described as benign by the Deafness Variation Database.^12^

Variant p.(Ser199Glnfs*9) is a previously unreported frameshift variant detected in two study participants, in one of them as a previously unreported case of compound heterozygosity with 35delG (p.[(Gly12Valfs*2)(;)[(Ser199Glnfs*9)]). Like p.(Ile196Thr), this variant also changes the amino acid sequences of the last transmembrane domain of connexin 26. None of the two participants with this variant had a family history of deafness or consanguinity. Another variant has been previously detected at position 199, the missense p.(Ser199Phe) variant, which is described as pathogenic in the homozygous state by the Deafness Variation Database.^12^

## Conclusion

The study of gene GJB2 in this multiethnic population demonstrated variants previously undescribed or rarely described. This suggests that a complete evaluation of this gene through gene sequencing, rather than just identifying mutation 35delG, should be the rule. Furthermore, new studies, investigating other genes, such as GJB6, must take place in order to identify the etiology of hearing loss of these patients.

## Conflicts of interest

The authors declare no conflicts of interest.
